# Complete Genome Sequence of the Fish Pathogen *Yersinia ruckeri* Strain SC09, Isolated from Diseased *Ictalurus punctatus* in China

**DOI:** 10.1128/genomeA.01327-14

**Published:** 2015-01-08

**Authors:** Kai-yu Wang, Tao Liu, Jun Wang, De-fang Chen, Xue-jing Wu, Jie Jiang, Jia-xing Liu

**Affiliations:** a College of Veterinary Medicine, Sichuan Agricultural University, Ya’an, Sichuan, Zhōngguó, China; b Key Laboratory of Animal Disease and Human Health of Sichuan Province, Sichuan Agricultural University, Ya’an, Sichuan, Zhōngguó, China; c Department of Aquaculture, College of Animal Science & Technology, Sichuan Agricultural University, Ya’an, Sichuan, Zhōngguó, China

## Abstract

Yersinia ruckeri SC09 is a Gram-negative bacterium isolated from a moribund Ictalurus punctatus collected in Jianyang, China. Here, we report the complete genome sequence of this microorganism to facilitate the investigation of its pathogenicity and to reevaluate its taxonomic position.

## GENOME ANNOUNCEMENT

Yersinia ruckeri is a Gram-negative rod-shaped bacterium causing enteric redmouth disease (ERM) in various fish species, leading to serious economic losses in aquaculture. The bacterium was initially isolated from diseased rainbow trout in the United States as early as the 1950s (1). Since Y. ruckeri was first reported, knowledge of the hosts and geographic ranges has increased. It has been isolated in many countries around the world, ranging from different hosts such as rainbow trout, carp, Ictalurus punctatus, sturgeon, burbot, and perch (2). Currently, it is one of the most important infectious diseases in Ictalurus punctatus aquaculture in China (3). Nevertheless, the genetic background of Y. ruckeri is still unclear, although a draft genome sequence of a motile O1b Y. ruckeri isolated from Atlantic Salmon in Chile has been reported recently (4). In addition, the taxonomic position of Y. ruckeri has not been accepted generally, and it was supposed to be reevaluated, perhaps as a new genus within the *Enterobacteriaceae* (5–7). So far no Y. ruckeri have been sequenced at the complete genome level. So, for the purpose of systematic research in Y. ruckeri, we report a complete genome sequence of Y. ruckeri isolated from Ictalurus punctatus in China.

Genomic DNA of Y. ruckeri was extracted using a TIANamp bacteria DNA kit (Tiangen Biotech CO, LTD.). The Illumina HiSeq2000 and MiSeq platforms were used to construct 2 different genomic DNA libraries according to the manufacturer’s instructions. Long-insert (2-kb to 6-kb) libraries were sequenced by the paired-end mode using the Illumina HiSeq2000, and short-insert (500-bp) libraries were sequenced by the paired-end mode with the Illumina MiSeq. Filtered paired-end reads (3,002 Mb in total) were obtained, giving 769-fold coverage of the genome. The sequence reads were assembled into one chromosome using the SOAPdenovo alignment tool (version 2.04). The chromosome contains 6 large scaffolds, including 31 contigs. So far, the interscaffold gaps have not been closed.

The completed genome of the Y. ruckeri strain comprises 3,923,491 bp with 47.45% GC content. The total number of predicted genes is 3,651, with a total length of 3,307,170 bp, and their percentage in the total genome is 84.29%. In the analysis of gene function of Y. ruckeri, the KEGG, COG, Swiss-Prot, TrEMBL, NR, and GO databases were used for function annotation. Meanwhile, the ARDB, CAZy, PHI, and VFDB databases were used for pathogen analysis. In addition, there are 137 interspersed repeats (IR) with 10,565 bp and 222 tandem repeats (TR) with 23,928 bp in the genome, respectively. We have also used rRNAmmer, tRNAscan, and Rfam to identify noncoding RNA (ncRNA), including rRNA, tRNA, and sRNA genes, respectively. In addition, the genome contains prophages, genomic islands (GIs), and one clustered regularly interspaced short palindromic repeat (CRISPR).

### Nucleotide sequence accession number.

The complete genome sequence of Y. ruckeri CH09 was deposited at GenBank under the accession no. JRWX00000000.
